# Urgent need hybrid production - what COVID-19 can teach us about dislocated production through 3d-printing and the maker scene

**DOI:** 10.1186/s41205-020-00090-5

**Published:** 2020-12-07

**Authors:** Sascha Hartig, Sven Duda, Lennart Hildebrandt

**Affiliations:** 1 Helmut Schmidt University, Institute of Production Engineering, Holstenhofweg 85, Hamburg, 22043 Germany; 2 Hospital of the German Armed Forces, Department of Neurosurgery, Lange Strae 38, Westerstede, 26655 Germany

**Keywords:** 3D-printing, Personal protective equipment (PPE), In-house production, Maker movement, COVID-19, Design in the loop

## Abstract

**Background:**

The COVID-19 pandemic has led to large-scale shutdowns in society. This resulted in global supply bottlenecks for medical protective equipment. The so-called Maker Movement recognized this emerging problem early on and, with the help of additive manufacturing (AM), began developing and manufacturing half masks or face shields as personal protective equipment (PPE). This knowledge has been made available in many places in form of open source product data, so that products could be adapted and improved, saving development time.

**Methods:**

This production and innovation potential has been taken up and professionalized by the authors of this article. By means of a proof-of-principle we provide an overview of the possibility and successful unique introduction of a so-called professional “hybrid production” in a micro factory using 3D-printing at the place of greatest demand in a hospital by medical personnel to produce their own PPE. Furthermore the learning process and future benefits of on site 3D-printing are described.

**Results:**

Our proof-of-principle successfully showed that the allocation of 3D-printing capabilities in the hospital infrastructure is possible. With assistance of the engineers, responsible for product design and development, the medical staff was able to produce PPE by means of AM. However, due to legal uncertainties and high material and production costs the usability is severely limited.

**Conclusions:**

The practical research showed that a complete implementation of the concept and the short-term establishment of a 3D-printing factory for the autonomous supply of a hospital with PPE was not feasible without further efforts. Nevertheless, it has enabled the medical staff to use AM technologies for future research approaches.

## Background

On December 31 2019, the WHO China Country Office was informed about unusual pneumonia in Wuhan (Hubei Province, China), the etiology of which was unknown at that time [1]. A few days later it became known that the lung disease was caused by a novel corona virus [2]. Since then, the virus has spread rapidly internationally, and the number of cases has increased exponentially. Therefore, the World Health Organization (WHO) has classified the outbreak as a pandemic since March 11 [1]. In Europe alone more than 1.3 million people have been verifiably infected with COVID-19 [3]. Due to the very high number of cases, some countries have meanwhile experienced supply bottlenecks for personal protective equipment (PPE) or other medical equipment[4], which is why new solutions to overcome the crisis and reduce the risk of an infection were sought (e.g. AM of short supply test equipment [5] or during the hackathon “#WirVsVirus” of the Federal Republic of Germany [6]). The so-called maker community therefore began early on to develop and build makeshift protective equipment (e.g. face shields[7] or breathing masks[8]). The wide-spread use of 3D-printing and the efforts of many professional scientists and non-professional hobbyists of the 3D-printing community have led to a large number of different open-source, non-certified face masks available for download and 3D-printing on a desktop 3D-printer [9–11]. Hospitals have also taken up these ideas, developed them further and produced and applied them locally [12]. In this article, the authors show a theoretical path for local, hybrid production of PPE and supplement this with practical insights from a proof-of-principle in cooperation with the German Armed Forces Hospital in Westerstede. Production within the hospital by hospital staff has several advantages. On the one hand, logistics chains and shipping routes can be minimized or even eliminated in times of poorly functioning logistics. On the other hand, specific product requirements and demands made by the users can be taken into account in order to improve the PPE. Our research deals with the integration of 3D-printers and their influence on the design development process, the advantages of in-house production and the insights of the medical staff on site. This point-of-care production strategy with on site printing and evaluation is the key feature of the study presented here.

## Methods

### Assembly of the 3D-Printing lab at the hospital

The aim of the experiment is the practical and theoretical analysis of how additive manufacturing (AM) can be implemented in a hospital, and how the cooperation between personnel and technical specialists can lead to innovative problem-solving products in the shortest possible time. To this end, the design process and the associated innovative approach to reduce time in product development by implementing AM capabilities at the user site and direct user feedback. The research was carried out as a cooperation between the German Armed Forces Hospital Westerstede and the Institute of Production Engineering of the Helmut Schmidt University in the period from March to April 2020. At the beginning, the needs for PPE, which can be covered by AM, were analyzed. The aim was primarily to supply the German Armed Forces Hospital Westerstede and subsequently to equip the affiliated cooperation hospital Ammerland-Klinik. After a first screening of the products available in the community, first prototypes were produced at the Institute of Production Engineering in Hamburg. These were sent by post to German Armed Forces Hospital in Westerstede, where direct user feedback was provided. Within this process, the procurement of several Fused Filament Fabrication (FFF) printers was pushed forward. The purpose of these printers was to enable a first production as well as accelerate the design process through faster feedback. The integration of additive production facilities at the user’s site creates the possibility of rapid product development, which is called Agile Engineering [13]. The customer worked together with the designer on the functions in order to develop a suitable product.

### Rapid prototyping and evaluation phase

**Fig. 1:**
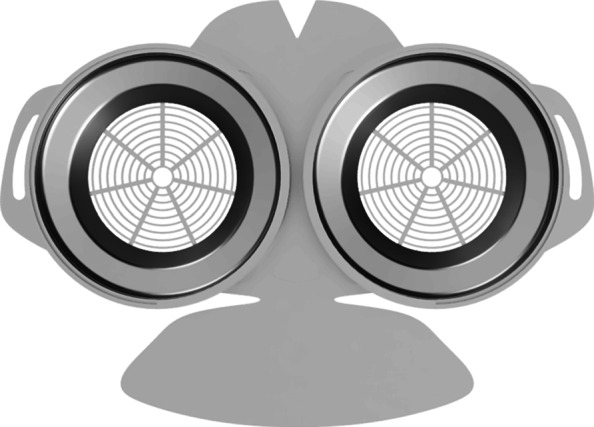
HSU/BwKWST FM V1. The fist version of the developed face mask has an thread insert for aftermarket FFP2 filters

**Fig. 2:**
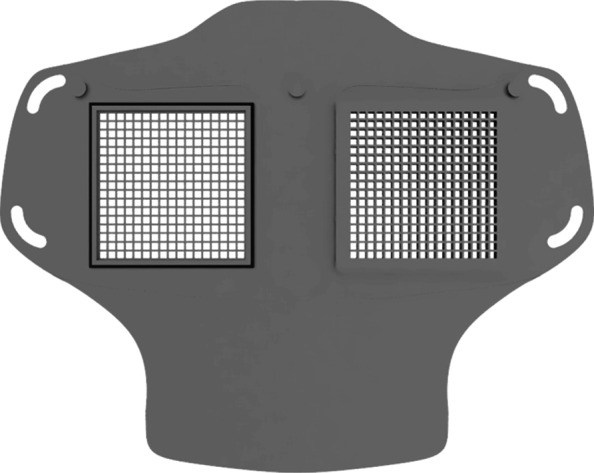
HSU/BwKWST FM V2. The second iteration has divided attachment possibilities for the rubber bands, as well as own filters, which can be made in own production with 25 *cm*^2^ filter material

**Fig. 3:**
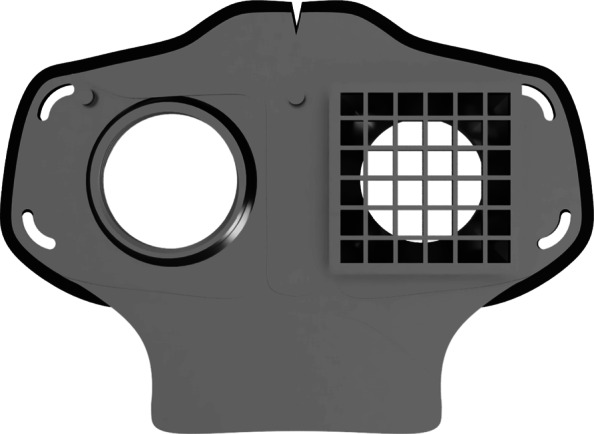
HSU/BwKWST FM V3. The third iteration has a sealing lip made of thermoplastic polyurethane, as well as a filter attachment that is screwed in for a larger filter surface

**Fig. 4:**
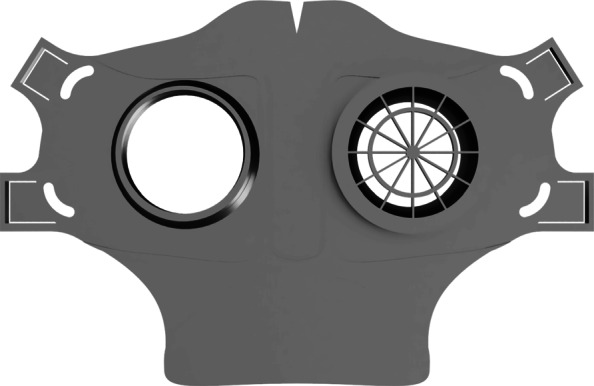
HSU/BwKWST FM V4. The final version has an improved holding system for wide elastics for better comfort. This design also dispenses with the sealing lip due to increased leakage

As part of our research novel face mask designs (HSU/BwKWST FM V1-4) were developed [14]. The different versions from one to version four are shown in Figs. 1, 2, 3 and 4. In the beginning, version one was used to try out a possibility based on purchased FFP2 filters. In the following iterations of the design, filters were produced additively, which are planned to be equipped with cut filter material. With regard to filters, FFP3 filter fleece was considered to be incorporated to the filter housings of the masks. Therefore a German manufacturer for FFP3 qualified fleece was contacted during our study and FFP3 fleece was provided for research purpose only.

**Fig. 5:**
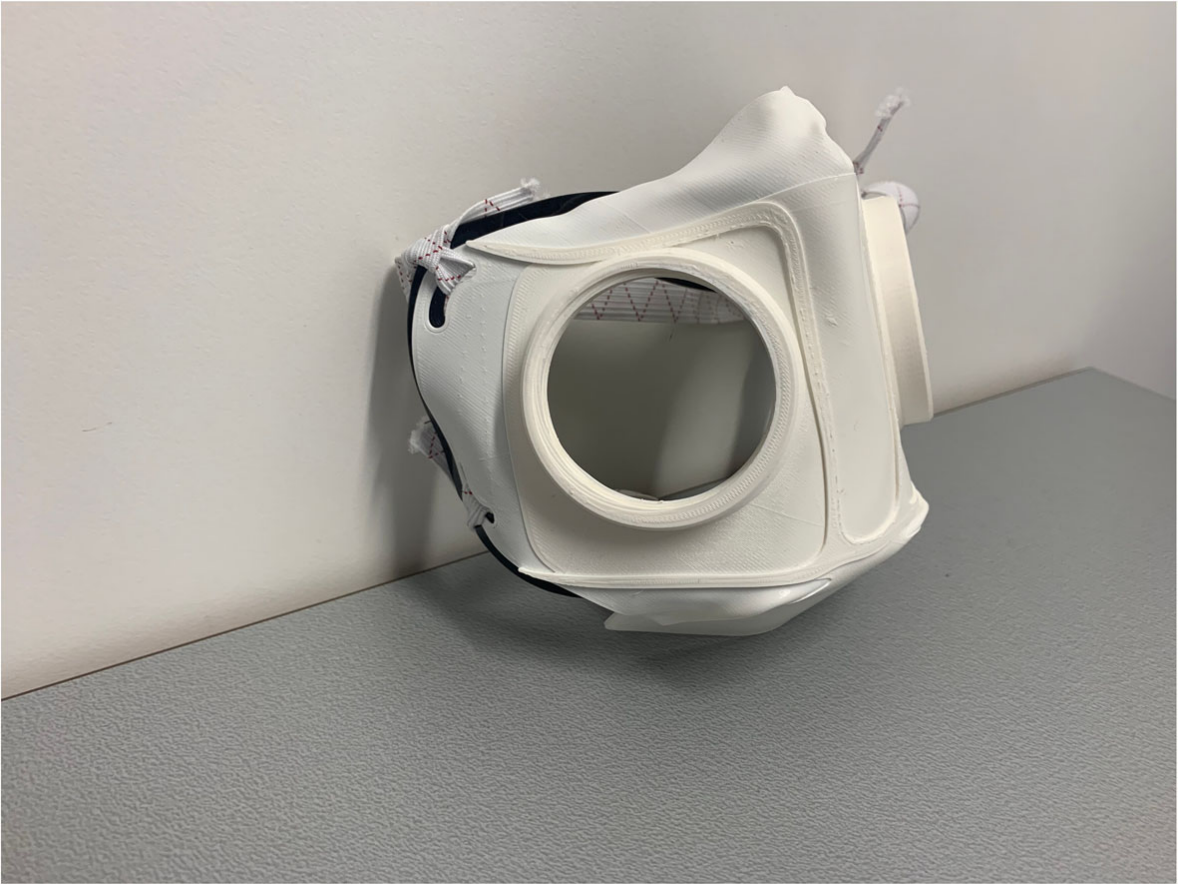
Side view HSU/BwKWST FM V3. Thermoformed face mask in version three with elastics and TPU sealing lip, black

The adaptation of the face mask to the own face shape was done by a thermoforming process. For this purpose, a hot air gun on the lowest setting was used. By heating PLA to 50 ^∘^C, the glass transition temperature can be exceeded and thus the material can be plastically deformed. The process of cyclic heating and fitting was carried out until a comfortable and tight fit was achieved. The use of hot water, as used for the adaptation of mouthgards in boxing, also facilitates viable fitment results. Such a fitting process is shown in Fig. 5 for the HSU/BwKWST FM V3.

### Theoretical setup of a printing farm

**Fig. 6:**
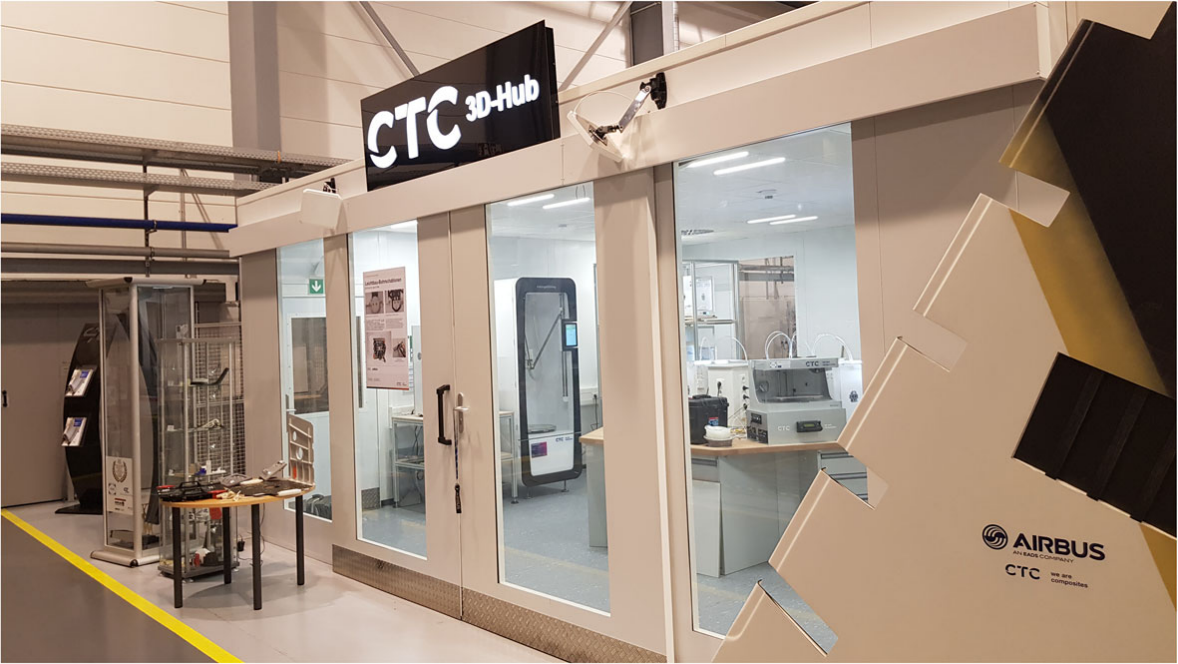
Printer Farm at CTC GmbH (an AIRBUS Company) in Stade, Germany. The printer farm at the CTC GmBH in Stade is a good example how a print farm can be build and what features can be implemented

Print farms are state of the art and have been in consistent development over the last years. The numbers of printers in the printing farm can range from 5 to 10 to over 1000 for example at PRUSA Research[15]. Printing farms are most commonly used for in-house production for supporting the design, research and engineering team as well as for consumer product production [16]. Due to the small space required, the printers are usually arranged on shelves to accommodate as many printers as possible. Various aspects must be taken into account when deciding on the installation place. These include temperature development, power consumption, material consumption, ventilation and extraction of harmful substances etc. Aspects such as consumption and heat development increase linearly with the number of printers. For this reason, if the number of printers exceeds a certain level (process specific), the rooms must be specially equipped for the installation of a production facility. Such a facility, specifically designed for the installation and use of 3-D printers, is shown in Fig. 6. The 3-D Hub of the company CTC GmbH, an AIRBUS company, provides a safe place with active extraction for operation. In addition, the consumption and whereabouts of raw materials are documented fully automatically.

In the following a theoretical approach was conducted to evaluate the possibility of autonomous production of PPE within a hospital environment. Therefore, the demand for face masks per day of the military part of the civil-military cooperation hospital was calculated by the number of clinical personnel. Two face masks per person were planned. At the given time 407 employees were working at the hospital. This results in a total of roundabout 814 masks per day. A production time of 5 hours per mask was assumed [14].

## Results

### Assembly of the 3D-Printing lab at the hospital

**Fig. 7:**
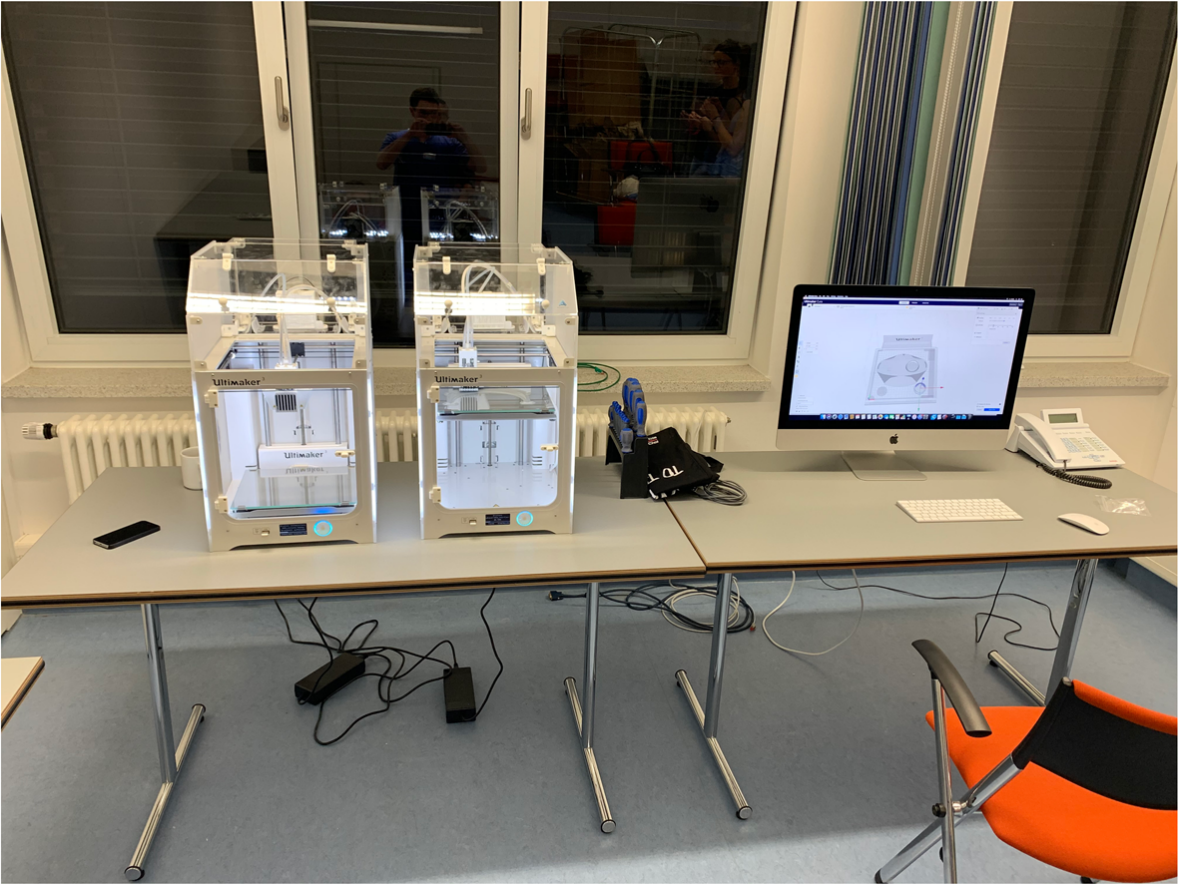
Early test setup. This was used for experiments, development and in-house production at the German Armed Forces Hospital in Westerstede

As early as in March 2020, at the very beginning of the COVID-19 pandemic in Germany, a 3D-printing lab was established as part of the 3D-Printing work group of the Neurosurgical Department at the German Armed Forces Hospital in Westerstede. For printer selection local availability, simplicity in use and maintenance were the main criteria considered. The setup, including four Ultimaker 3 Printers (Ultimaker, Geldermalen, Netherlands), was installed in close cooperation with the engineers of the Laboratory for Manufacturing Engineering at the Helmut Schmidt University, University of the German Federal Armed Forces Hamburg. The first stage of this test setup is shown in Fig. 7. The first setup consisted of two Ultimaker S3 and the necessary computer. Interdisciplinary video conferences were used to coordinate the installation process and early production attempts.

### Rapid prototyping and evaluation phase

During the crisis, the integration of 3-D printers on the side of the medical staff allowed an accelerated product development cycle to be carried out. With this method of product development, the time from the first drafts to the finished product can be considerably reduced by the production of prototypes at the user’s site by the user and the simultaneous performance of a direct product evaluation. In the experiment carried out by the authors, a time saving of three days per iteration loop was achieved by eliminating the process of transporting the prototypes to the customer. Afterwards the lab was used to develop various 3D-printed devices for personal protection in the medical sector, i.e. face masks, face shields and safety goggles. Therefore, the principle of Agile Engineering was applied as described earlier in this paper. After on site 3D-printing in Westerstede, a thorough evaluation of the printed protective gear took place. Suggestions for improvement were collected and referred to the designing engineers. The prototype files were tailored to the requirements determined by the physicians and sent back for reprint. Until now, more than 200 devices (100 faceshields made from PETG, 25 face masks made from PLA, 25 face masks made from TPU, 50 goggles made from PLA) for the personal protection against SARS-CoV2 have been printed in Westerstede. Initially an approach to fabricate masks prepared for available FFP3 filters was followed. The concept was quickly rejected due to the difficulty of sourcing and the weight of these filters. Furthermore, the limitation to size and thread of a filter type is not appropriate due to the logistical bottlenecks paired with the social buying behaviour during the first weeks of the crisis. Recent data from our research group showed that all of the tested, 3D-printed respirators evaluated in our lab do not qualify as medical protective gear as they fail to achieve the N95 standard for face masks [14]. As most of the available masks with the FFP3 fleece applied do not qualify for a use as medical protective gear due to small filter areas, a further testing of the filtering capacity was not conducted in our study [14].With respect to disinfection the physicians found that a warming of masks previously thermoformed to the users face using the thermoplastic material properties, resulted in a reversion to the original, fold-able (i.e. flat) form. With the absence of creases the masks were more easy of access for disinfection procedures. As the masks failed to achieve the N95 standard they were not routinely used in our hospital and further research on disinfection protocols was not conducted with face masks.

### Theoretical setup of a printing farm

Theoretically 4.8 masks can be produced per printer per day in 24-hour operation. If these 814 masks are to be produced daily, 170 printers are required. These 170 printers in turn require a floor space of 42.5m ^2^ calculated on a needed space of 0.25m ^2^ per printer. To accommodate them in one room, two levels could be arranged on shelves. This corresponds to 42,5 shelf meters of printers.With an average power consumption of 100 W during operation (here Prusa i3Mk3s), approximately 17 kW of supply power is required for 170 simultaneously running printers. This must be considered in the preparation of the printer farm. In the last iteration stage of the mask called HSU/BwKWST FM V4, 97 g of material are used per print. This leads to a daily consumption of approximately 79 kg polymers. Due to the number of machines depending on manufacturer and model, the investment requirement for the printers is shown in Table 1.

**Table 1:** Investmentcost for printers only

	Prusa Mini	Prusa i3Mk3s	Ultimaker S3
Unit price	379€	1000€	4000€
170 pieces	64430€	170000€	680000€

**Fig. 8:**
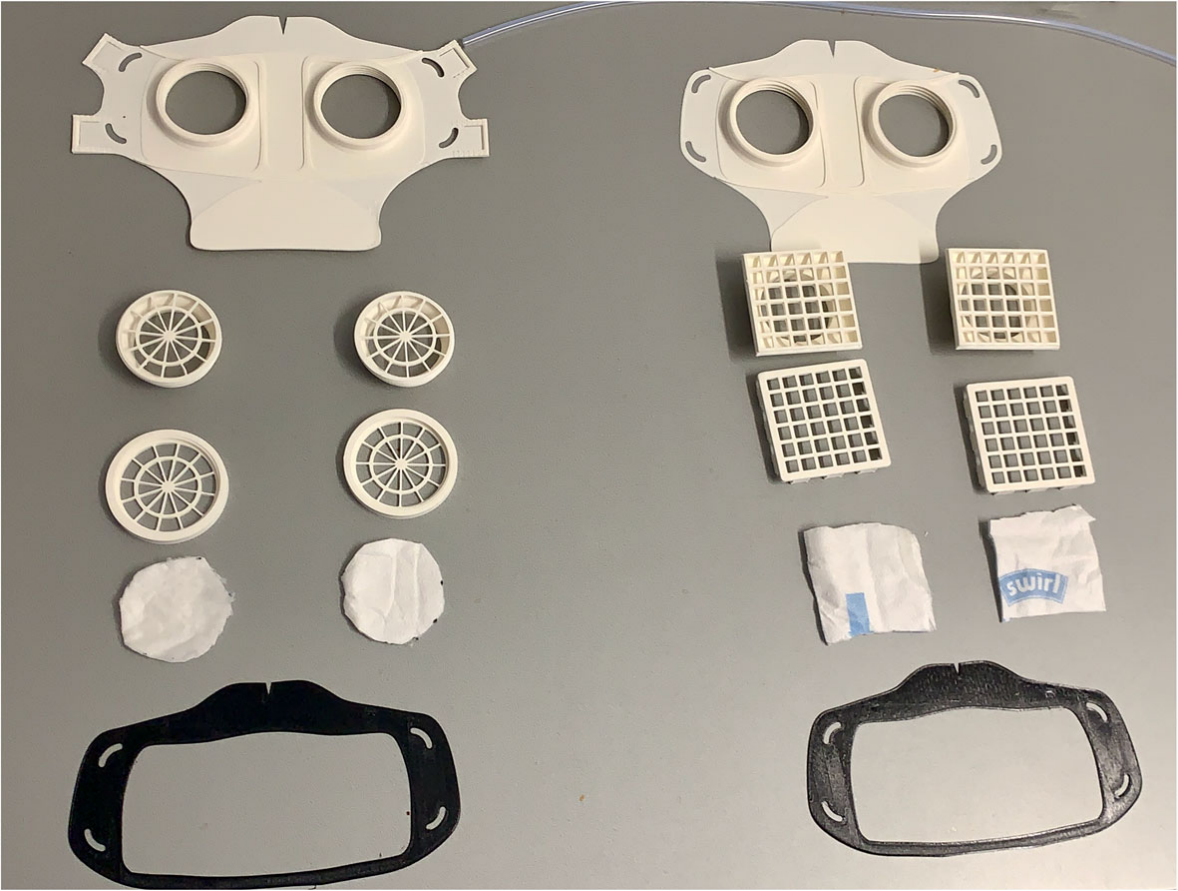
Comparison of all parts HSU/BwKWST FM V4 left and V3 right. From top to bottom the following parts are shown: PLA Mask body, PLA two-piece filter inserts, temporary filter mat cuttings, TPU sealing lip

**Fig. 9:**
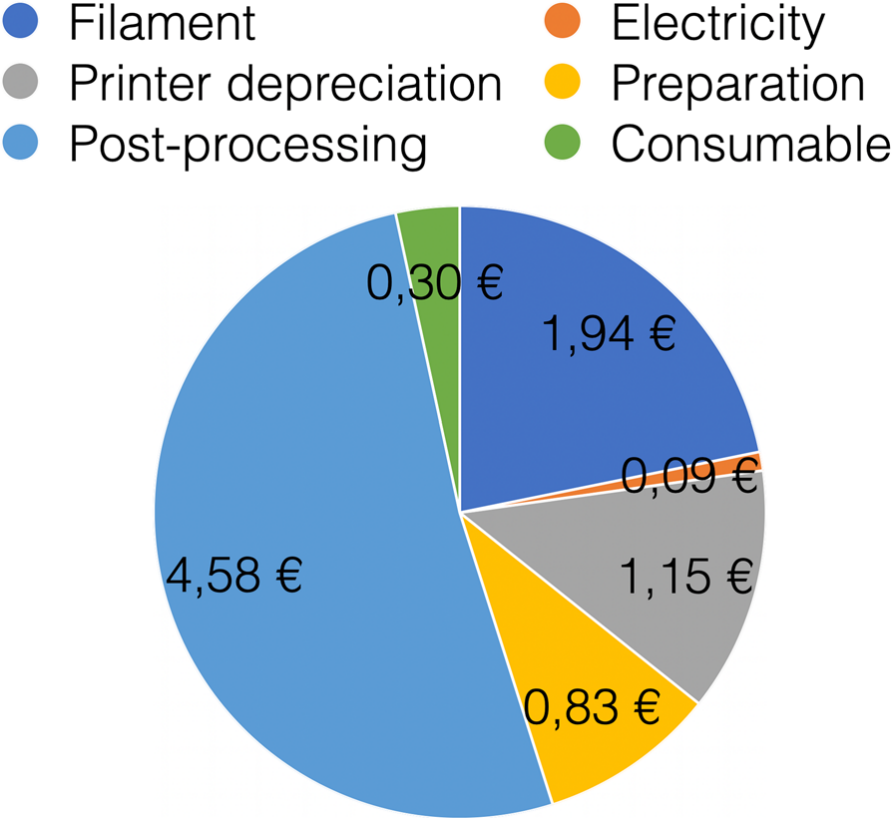
2D Pie diagramm showing the cost breakdown of one HSU/BwKWST FM V4. The largest proportion of costs is caused by post-processing followed by filament, preparation, consumables, depriciation of the printer and electricity

For material costs, a daily requirement of about 1580€ can be estimated when printing 814 masks needing 79kg of polymers with a filament price of 20€ per kg. These costs only include the pure material and equipment costs for the production of the necessary face masks. In addition besides the estimation of the total costs, filter material, rubber bands and labour costs have to be added. All needed parts are shown in Fig. 8 for V3 and V4. The manufacturing costs of one mask were calculated using the open source software by Hermann [17]. With the basic values, listed in Table 2, a unit price per mask of 9,78€ was calculated. The distribution of the costs is shown in the diagram in Fig. 9.

**Table 2:** Basic parameters for the direct cost calculation per mask

Basic parameters	Characteristic value	Costs
Printer	Prusa i3Mk3S	1000€
Filament	PLA	20€/kg
Labour Costs		50€/h
Energy Costs		0,28€/h
Failure rate	10%	
Consumable	Filter material	0,17€
Consumable	Elastic band	0,13€

**Fig. 10:**
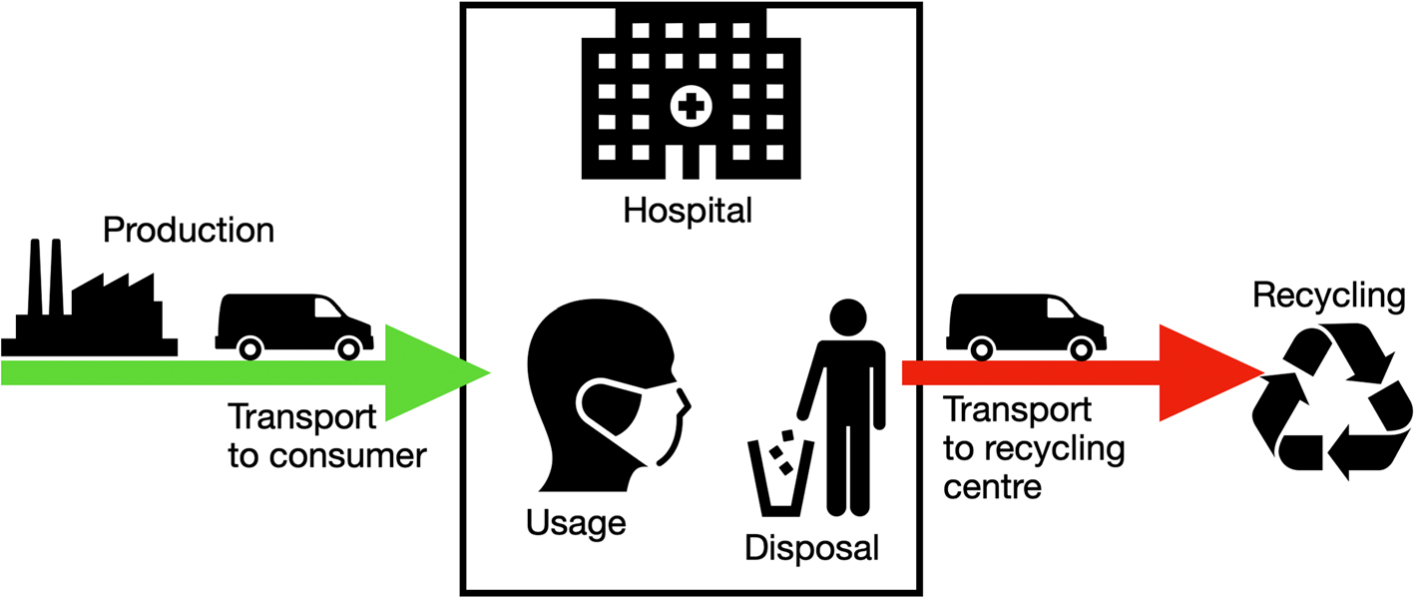
Life cycle of a medical device according to current industry standards. The medical equipment is manufactured within an industrial company. The product is then transported to the hospital and stored there until it is used. After use, it is transported to the waste collection system as medical waste

**Fig. 11:**
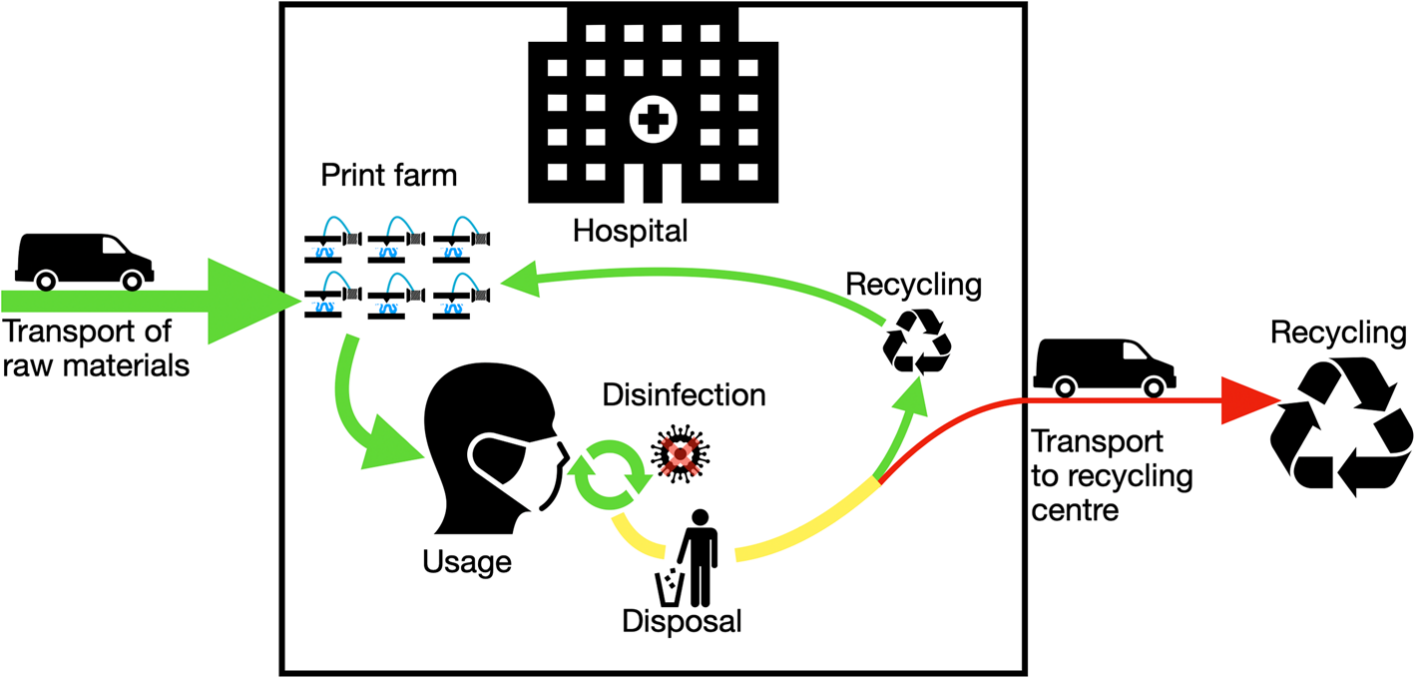
Altered life cycle for a medical device with in-house production and disinfection or recycling after usage. Only the raw material for production will be delivered to the hospital. After production and the subsequent quality assurance, the products are ready for use. After usage, the products undergo disinfection and, if desired, are reused. Otherwise, the waste is recycled or disposed

As the procurement of materials and the costs incurred are subject to strong fluctuations during the crisis, the cost calculation carried out must be viewed critically. There were deviations in the range of 1000% in the rubber band consumables alone. The working hours are estimates based on the first prototypes produced and can therefore deviate greatly from a possible mass production. The calculation basically shows a basic estimation of the cost range of AM for a face mask. However, raising this budget has to be viewed critically in a time of crisis, even the German state does not intend to contribute to material costs, although the expenses for protective equipment have increased significantly [18]. In Fig. 10 the theoretical linear life cycle of typical PPE like a face mask is shown. The mask is manufactured within an industrial company. The product is then transported to the hospital and stored there until it is used. After use, it is transported to the waste collection system as medical waste. With this possibility of disinfection, shown in the altered life cycle in Fig. 11, the second goal of supplying the civil part of the civil-military cooperation hospital could be achieved. An additional staff of 1500 people is employed here. As one mask is in use, one is in reprocessing and another one should be provided for short-term change, three masks per person are required. Thus, together with the German Armed Forces Hospital in Westerstede, 5721 disinfected reusable masks are consequently required per day. By reusing the masks, the number of printers can be reduced to 150 and therefore the investment cost for printers. The daily capacity with 4.8 masks of 150 printers is 720 masks. To produce the initial stock of masks, about 8 days in 24-hour operation are required. Assuming a mask failure rate of 10%, the printer farm must then produce 572 new masks daily. This means that 30 printers can be used for other purposes. Initially around 555 kg and then 55.5 kg of PLA are required daily for mask production. With the above mentioned material costs this corresponds to 11100€ and daily 1110€.

### Technical issues and lessons learned

During our proof-of-principle, several minor repairs, calibration and maintenance procedures were necessary and performed on site (i.e. defect y-switch, obstructed and defect nozzles, print head disassembly, x-y-calibration, z-offset calibration). The application of 3D-printing by the physicians themselves led to a thorough understanding of the processes involved in preparation and 3D-printing as well as material science. This resulted in more precise requests to the designers as the capabilities of the available printing principle and material properties were now taken into account. From then on, the users had an idea of which geometric forms are realizable using 3D-printing. Furthermore, the physicians experimented with different printing parameters to learn their impact on the 3D-printed part. Together with the on site fabrication of parts and their immediate evaluation by the users themselves, this gradual process has led to a fusion of the end user and developer role. The understanding of these production specific aspects contributed well to the advance of our Agile Engineering approach. From a legal point of view, on site production at the hospital was considered to possibly circumvent approval difficulties, as the non-certified PPE is not placed on the market but only printed for self-protection by the end users themselves with production capabilities. Within this field arises the problem of liability in case of damage caused by the product. Under German law, distributors and manufacturers assume product liability regardless of whether a disclaimer statement has been issued. The problem can potentially be avoided if the user himself produces locally. The user then bears the risk of wearing the PPE. In case of the COVID-19 pandemic, these strategies were also worked on to generate the greatest possible readiness of material. A review of the product and medical product law by legal advisors of the Medical Goes Additive network during their monitoring assistance of product development assessed this approach as inadmissible.

## Discussion

### Innovations from the maker movement to fight COVID-19

Makers are creative tinkerers who develop and build their own ideas and prototypes using decentralized infrastructure (e.g. FabLab, Makerspace) or appropriate local access to digital fabrication machines [19]. The term “making” refers to the design and manufacture of utility and consumer goods [20]. Makers are therefore considered to have enormous innovation potential. The whole movement (the so-called “Maker Movement”) can even be seen as an industrial revolution [21, 22]. However, the main objective is not the production of mass products but the production of consumer goods for the users themselves [23]. For sectors such as niche products, personalized consumer goods and small series, the movement is seen as having great potential [24].

During the COVID-19 pandemic, the potential and the possibility of local self-production of commodities was exploited by the community of Makers and has led to significant regional improvements in the supply situation. Various projects have resulted in a wide variety of products, some of which have even been made publicly available as open source via the Internet, allowing collaborative development and local production [25, 26]. The advantages of the open source dissemination of knowledge and data and the production of open source hardware based on this data become particularly evident in such crisis situations. For the user, the use of open source hardware offers lower acquisition costs, higher resource efficiency, faster and cheaper support from the community, significantly lower R&D costs and lower or no legal fees [27–29].

These are potentials that ideally support the rapid response to a global pandemic with regional resource shortages and have therefore been used in large scale during the COVID 19 pandemic. For the rapid dissemination of data, open source databases and platforms have been generated for different use cases (e.g. case numbers, drug analyses, test systems), which can be accessed by users [30–33].

According to WHO the virus primarily spreads by aerosols containing virus, which is why many open source hardware projects were essentially limited to reducing the risk of such an infection [34, 35].

A well-known representative for this is the “DIY-Community-Mask”, which can meanwhile often be found as makeshift respiratory protection and to reduce the emission of droplets when breathing, coughing or sneezing [36].

In addition, half masks with replaceable filters have also been developed by the maker community, which above all should reduce the wearers infection risk [35]. Under the Italian “Open Source Mask” project, Makers have collaborated internationally, and together they have developed and tested a total of 35 half-mask models and made them available for download on the project website [37]. With the help of the files, a half-mask can be produced locally and according to requirements using appropriate digital fabrication machines (in particular through AM using FFF). Only the filter elements (mostly so-called high efficiency particulate air filters) have to be procured.

But the open source half masks have in common that they are not certified PPE and should only be used in exceptional cases [14]. They do not replace the certified protection of other measures, but are intended, as in the case of the COVID-19 pandemic, to enable a larger number of people to protect themselves to a certain extent. However, they should only be used if certified protection measures are not available.

### Professionalization through local hybrid production

To overcome the identified problems the idea and possibility of half-mask production has been taken up and professionalized by the authors in order to enable medical personnel to print or pick up a half-mask locally at any time. For this purpose, Open source product data and designs that were distributed via the Internet were tested and evaluated. The designs were then further developed and adapted to the needs of the hospital. By using open source hardware, the development cycle was significantly shortened, as it was possible to build on existing global knowledge and to manufacture and test locally. As test and production environment a hybrid production facility has been set up in the Westerstede Military Hospital. Hybrid production aims to increase the added value of different stakeholders in a joint collaboration in one production capacity (so-called urban micro factory) [38]. In this research approach the application of hybrid production enables doctors and medical staff to produce their own professionalized half-masks in the hospital, which allows them to maintain their personal protection while participating in the development process by sharing feedback.

In addition, they learn how to handle digital manufacturing processes, which enables them to produce new products or disruptive adjustments according to the principle of bottom-up economy [39]. Conversely, sharing the designs and experiences creates added value for the maker community, which can use the corresponding design. In addition to in-house production, the hybrid micro factory in the hospital also has its own production team, which also produces half-masks for output and is available to answer questions.

From the point of view of a researcher with research activities in the application of 3D-printing in the medical sector, the COVID-19 pandemic led to far-reaching changes both in research fields and the conduction of this research. To this point AM was mainly used to fabricate models for patient education, patient specific implants, surgical instruments, surgical planning and simulation [40, 41]. Research was mainly conducted with an individual approach in different medical disciplines and research groups. In contrast to the segmentation of image data, which is in our case done at the hospital, print job preparation and execution of the print job are done by service providers or associated technical research labs [42]. With the onset of serious supply concerns regarding PPE during the COVID-19 pandemic the activity of the Maker Scene led to a conversion of the medical 3D-printing research activities from an individual approach to a global joint research activity with the objective of globally available open-source data to produce qualified PPE at the point of need [26]. The previously mentioned benefits of on-site production and evaluation led to the implementation of a 3D-printing architecture within our hospital. As opposed to former research activities the role of the medical researchers and engineers have changed in such a way, that the engineers now provide the print job data and the medical staff executes the print job. Lessons learned from this experiment can potentially lead to a further application of 3D-printing in the clinic for rapid tooling and prototyping attempts and research on patient specific implants or individual, tailored surgical instruments.

Our rudimentary approach at estimation of the effort of an autonomous PPE production at the point-of-need shows that there is a high resource requirement to cover the demand for masks. Disinfection of PPE is the key for minimizing consumption of resources and increasing the efficiency of in-house production. Through disinfection, equipment can be safely reused several times. Decontamination approaches for additively manufactured PPE were tested under laboratory conditions with COVID-19. A condensed list of the methods was compiled by PRUSA RESEARCH verified by laboratories and hospitals to ensure conclusive and reliable results [43]. In our theoretical approach application, for example, the proposed disinfection option with isopropanol (IPA) could be carried out in a bath. The masks are then dried, packed and prepared for the next use of the carrier with new filter inserts. Important in this approach is the correct storage of the masks, as well as the marking of the personalized masks, so that a direct assignment can take place and the personnel can reach the masks independently. To accomplish this, it is suggested that the mask be distributed and stored in sterile resealable bags after disinfection. A similar procedure is used for fire protection masks within the German Navy. Different filament colors are suggested for marking, so that each department or section gets its own color. This increases the effort within the production, but leads to an intuitive sorting possibility. Within the departments, the mask can then be assigned to its wearer with a permanently attached name plate on the mask, which is not affected by the disinfection process. Additionally, a print product recycling setup was investigated at the Institute for Production Engineering in Hamburg. With this being installed at the point of need and the site of use, 3D-printed PPE could in future be recycled instead of disinfected when necessary.

The COVID-19 pandemic shows, that communities and makers are willing to solve problems even with great effort. Nevertheless, the discussed legal aspects prohibit a premature execution of AM for PPE fabrication. As already mentioned, the product liability law and the legal basis of the marketer also applies in times of crisis. For products that are medical devices also standards as ISO13845, ISO10993 and ISO14971 have to be followed. This means that it is in principle not possible to use products that have been developed by the community and manufactured by medical personnel. Two additional parties are needed for this. On the one hand an expert from the field of medical product development as a mediator of the community, who is also responsible for documentation. As a fourth component, a company is needed which takes over the production of the products, taking into account the standards to be met. This is a step backwards from the basic concept, but a step forward in terms of possible deployment. Thus, due to the poor protective effect of PPE fabricated by AM technologies, high manufacturing and product cost and legal uncertainties an autonomous PPE production can not be easily implemented in the health care sector.

## Conclusions

In this research, the urgent need for PPE was addressed by implementing AM capabilities (hybrid micro factories) within a hospital. For this purpose, the authors gave an insight into the Maker Movement during the Corona pandemic and presented theoretical foundations and preliminary considerations for the implementation of hybrid production.

Even though the practical study showed that a complete implementation of the concept is not possible, it has enabled the medical staff to use the FFF process for the production of patient-specific products or surgical tools that meet the needs of the patients. With regard to PPE fabricated by AM, further product design changes and in-deep testing is necessary to evaluate the possibility of a future use of 3D-printed PPE in the health care sector. Afterwards a standardized testing protocol will have to be followed.

From a users point of view, the self-reliant application of 3D-printing facilitated becoming familiar with the possibilities and limitations of these techniques, which is absolutely necessary to exploit the capabilities of 3D-printing by means of hybrid production.

## Data Availability

Data sharing not applicable to this article as no datasets were generated or analyzed during the current study.

## Abbreviations

AM: Additive manufacturing
FFF: Fused filament fabrication
HSU/BwKWST FM: Helmut Schmidt University / Federal Armed Forces Hospital Westerstede Face Mask
PETG: Polyethylene terephthalate glycol-modified
PLA: Polylactic acid
PPE: Personal protective equipment
R&D: Research and development
SARS-CoV-2: Severe acute respiratory syndrome CoronaVirus type 2
WHO: World health organization
