# Correction to: Urgent need hybrid production - what COVID-19 can teach us about dislocated production through 3d-printing and the maker scene

**DOI:** 10.1186/s41205-021-00094-9

**Published:** 2021-02-08

**Authors:** Sascha Hartig, Sven Duda, Lennart Hildebrandt

**Affiliations:** 1 Helmut Schmidt University, Institute of Production Engineering, Holstenhofweg 85, 22043 Hamburg, Germany; 2 Hospital of the German Armed Forces, Department of Neurosurgery, Lange Strae 38, 26655 Westerstede, Germany

**Correction to: 3D Print Med 6, 37 (2020)**

**https://doi.org/10.1186/s41205-020-00090-5**

In the original publication [1] the figures and captions were incorrectly matched during an error in the publication process. This only affected the web publication and did not affect the PDF-publication. In this correction article the correct figure & caption combinations are published. The original article has been updated.

**Fig. 1:**
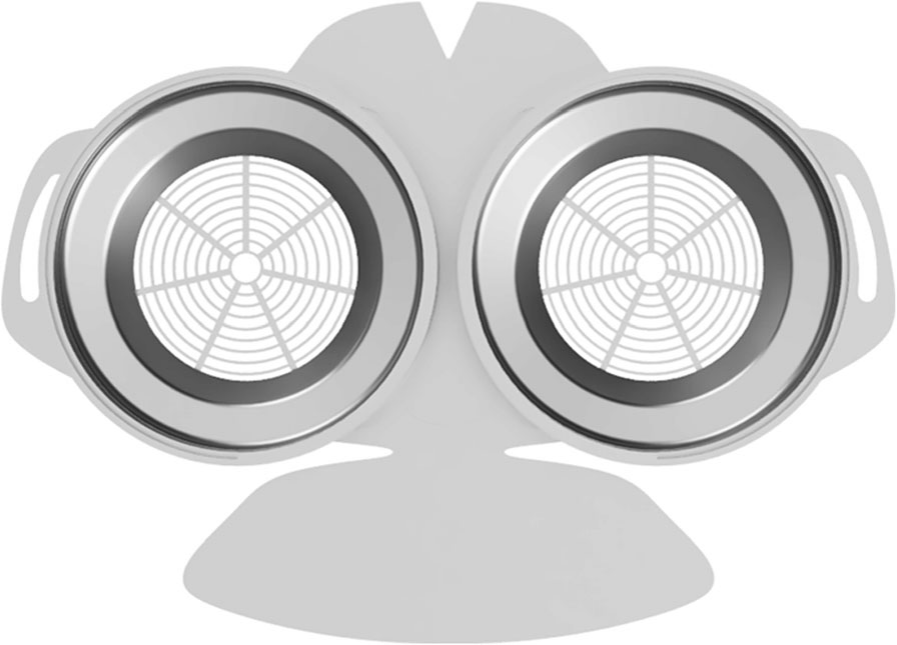
HSU/BwKWST FM V1. The first version of the developed face mask has a thread insert for aftermarket FFP2 filters

**Fig. 2:**
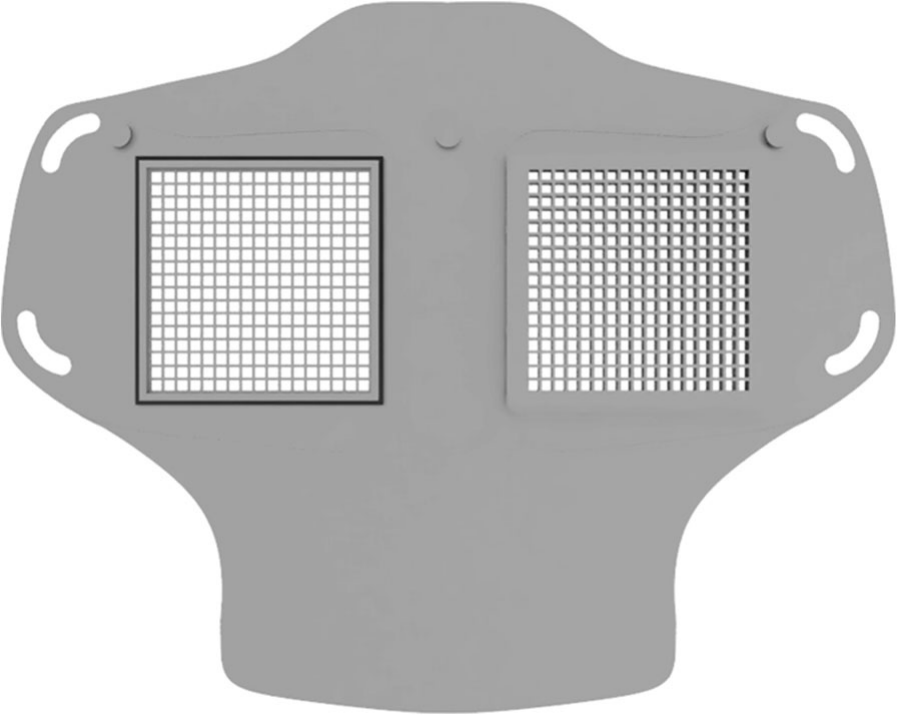
HSU/BwKWST FM V2. The second iteration has divided attachment possibilities for the rubber bands, as well as own filters, which can be made in own production with 25 *cm*^2^ filter material

**Fig. 3:**
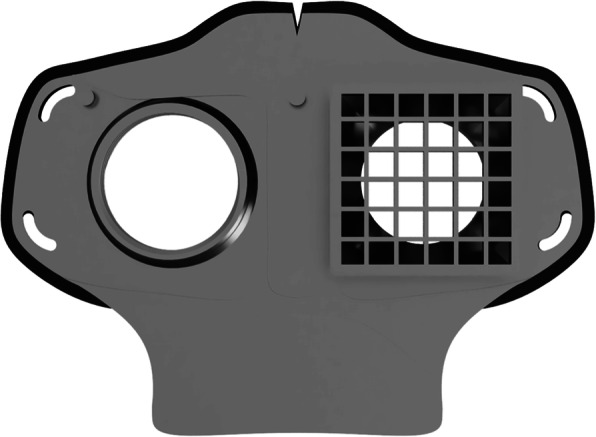
HSU/BwKWST FM V3. The third iteration has a sealing lip made of thermoplastic polyurethane, as well as a filter attachment that is screwed in for a larger filter surface

**Fig. 4:**
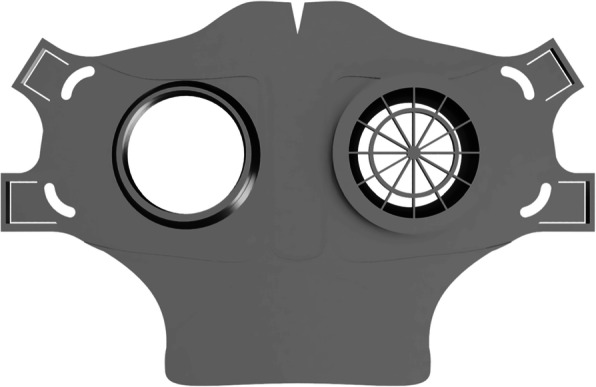
HSU/BwKWST FM V4. The final version has an improved holding system for wide elastics for better comfort. This design also dispenses with the sealing lip due to increased leakage

**Fig. 5:**
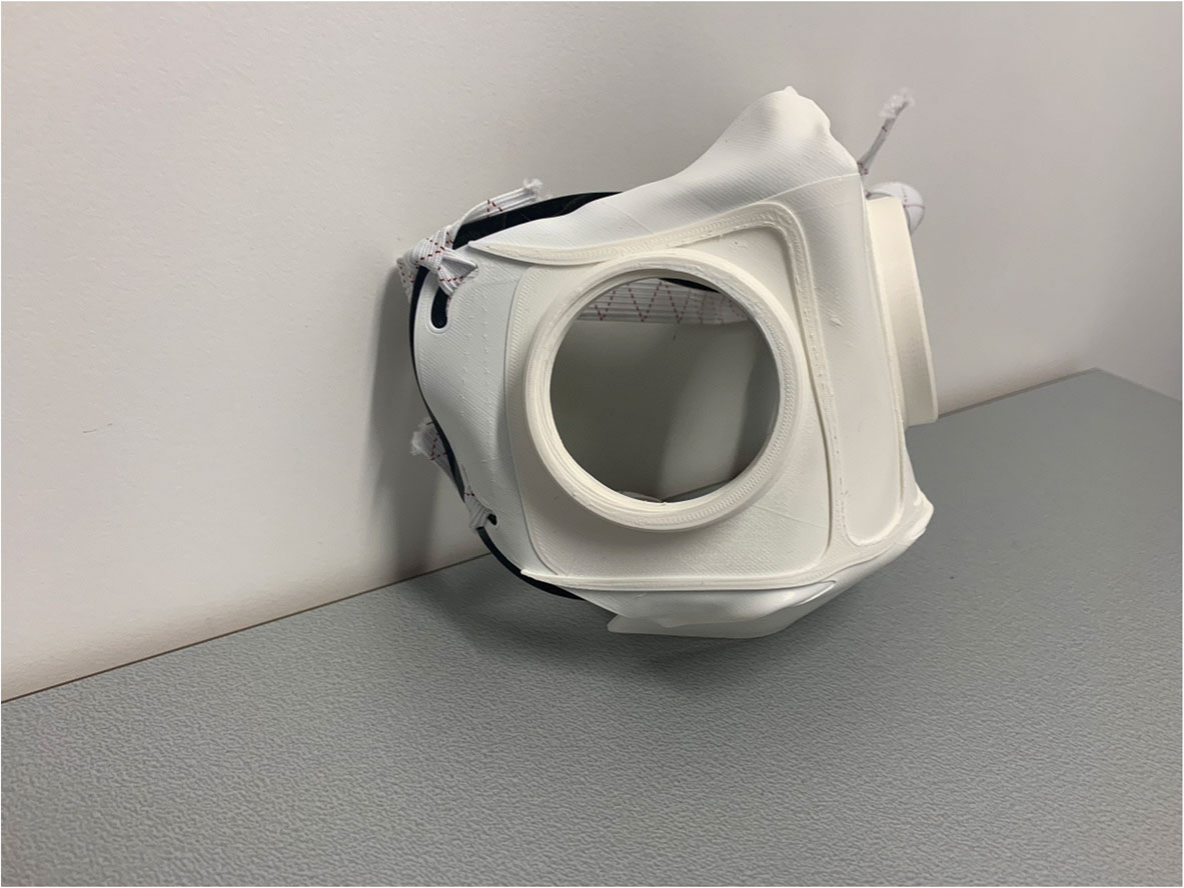
Side view HSU/BwKWST FM V3. Thermoformed face mask in version three with elastics and TPU sealing lip, black

**Fig. 6:**
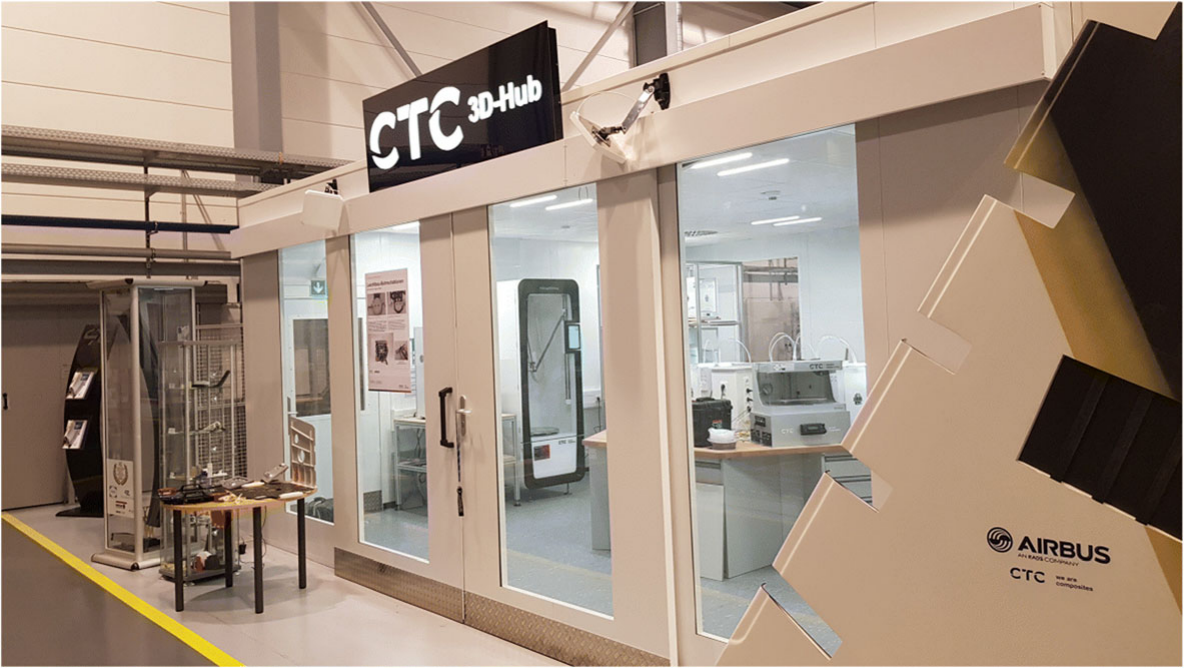
Printer Farm at CTC GmbH (an AIRBUS Company) in Stade, Germany. The printer farm at the CTC GmBH in Stade is a good example how a print farm can be build and what features can be implemented

**Fig. 7:**
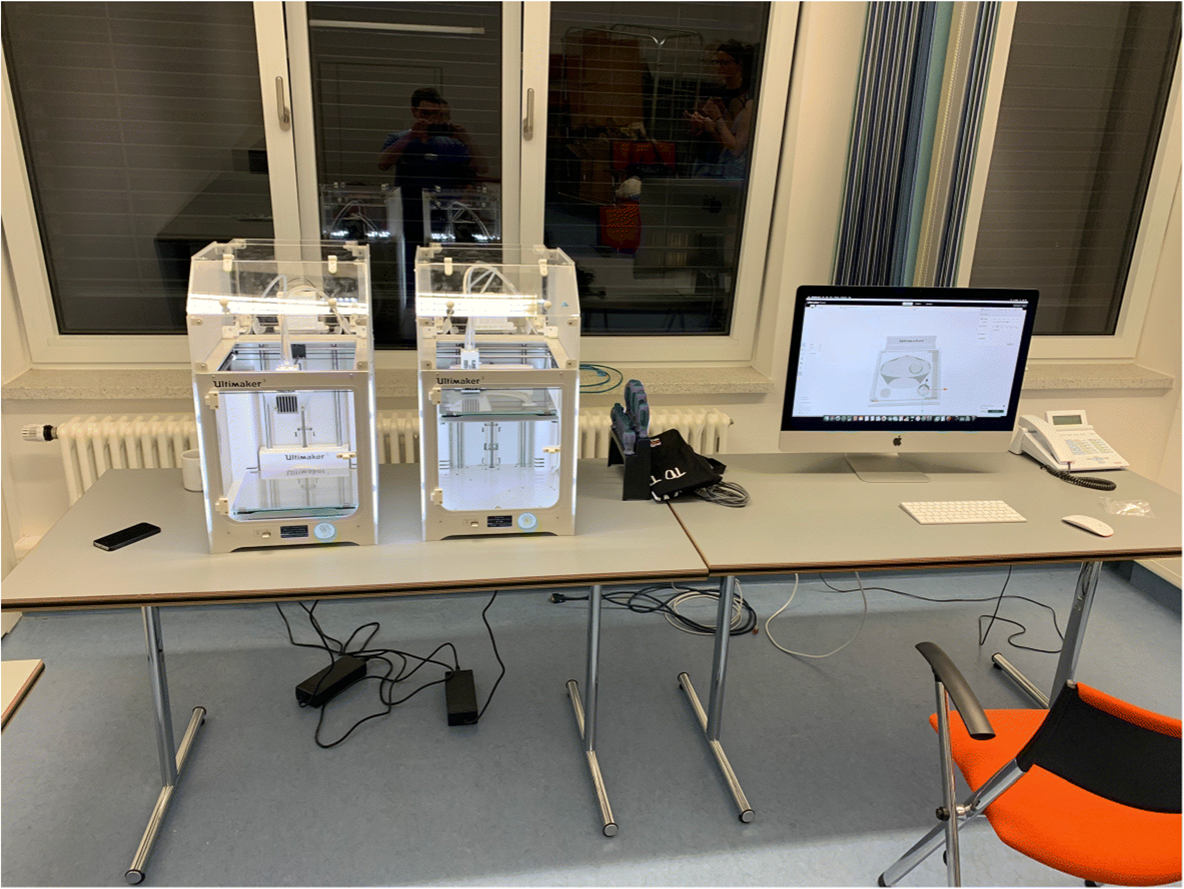
Early test setup. This was used for experiments, development and in-house production at the German Armed Forces Hospital in Westerstede

**Fig. 8:**
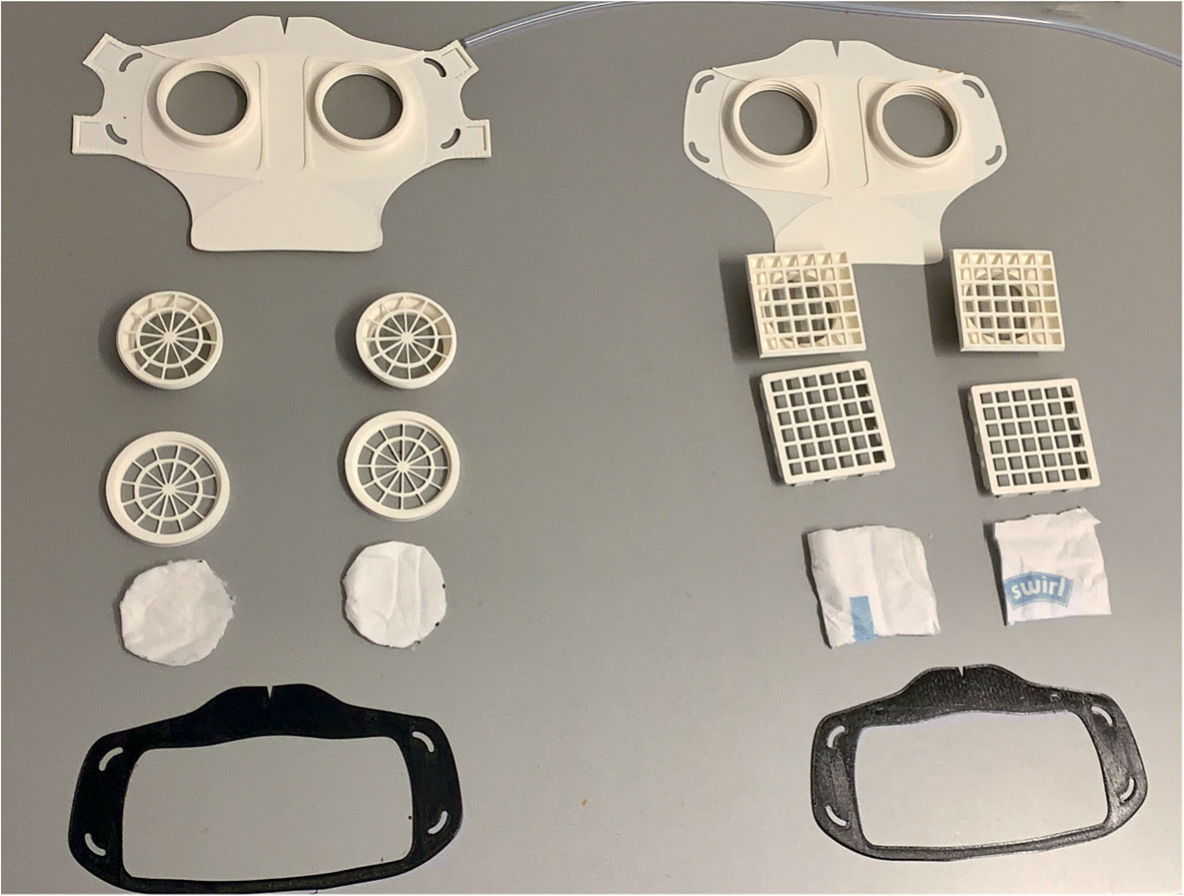
Comparison of all parts HSU/BwKWST FM V4 left and V3 right. From top to bottom the following parts are shown: PLA Mask body, PLA two-piece filter inserts, temporary filter mat cuttings, TPU sealing lip

**Fig. 9:**
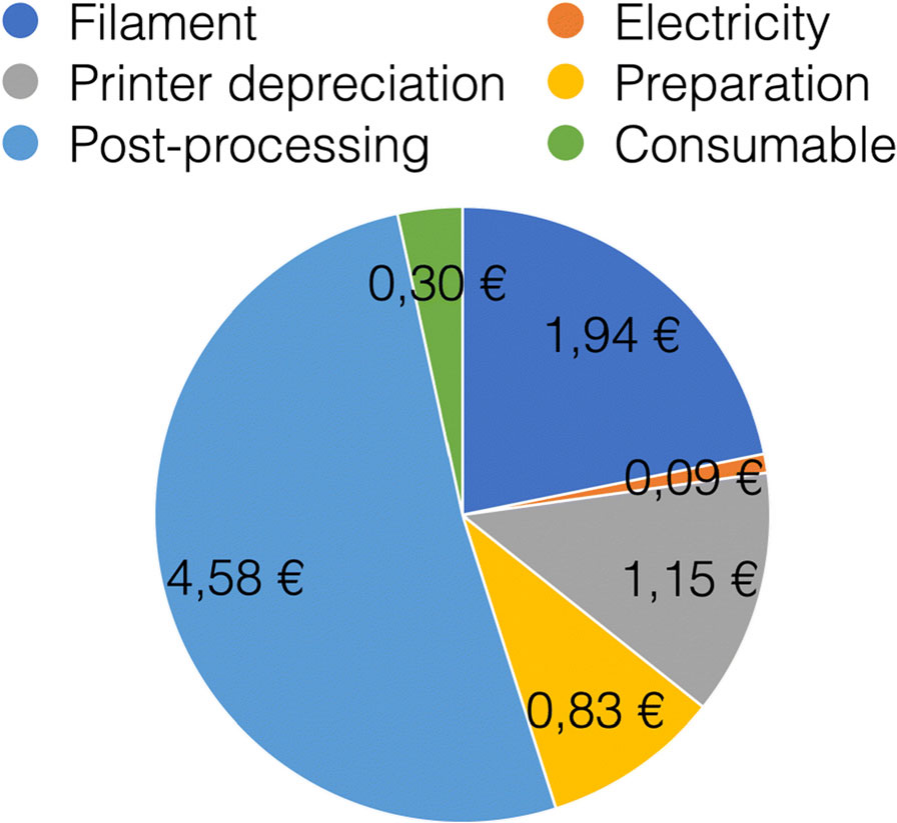
2D Pie diagramm showing the cost breakdown of one HSU/BwKWST FM V4. The largest proportion of costs is caused by post-processing followed by filament, preparation, consumables, depriciation of the printer and electricity

**Fig. 10:**
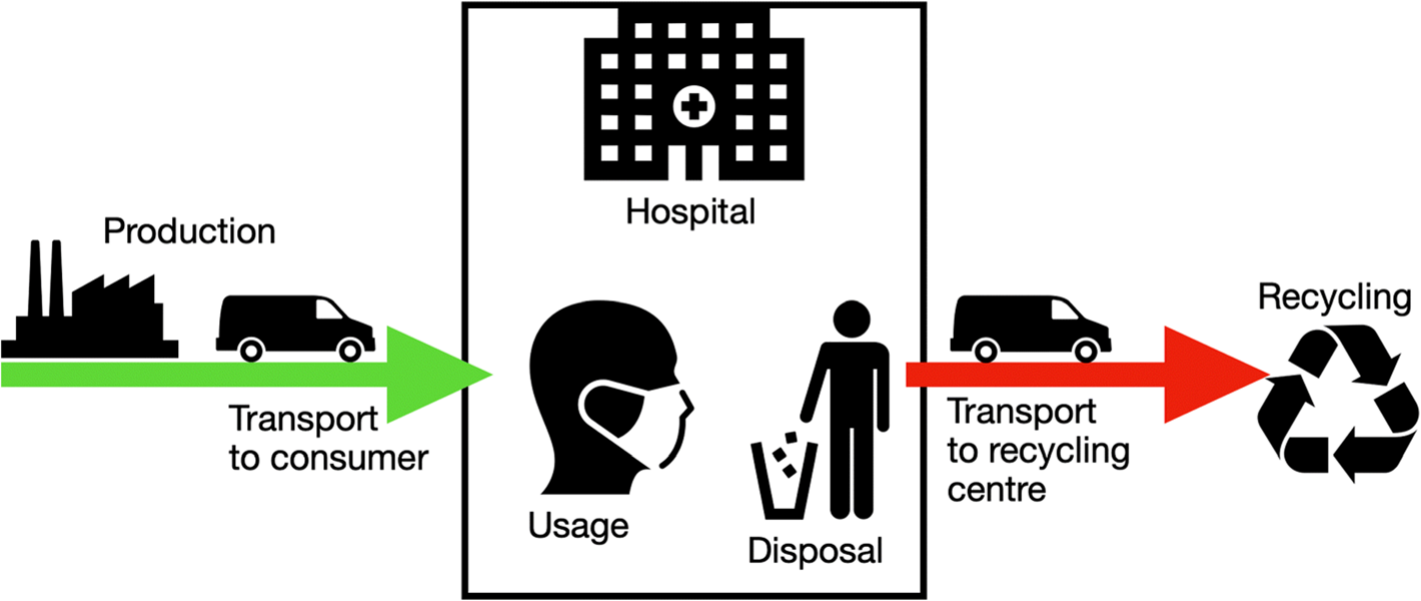
Life cycle of a medical device according to current industry standards. The medical equipment is manufactured within an industrial company. The product is then transported to the hospital and stored there until it is used. After use, it is transported to the waste collection system as medical waste

**Fig. 11:**
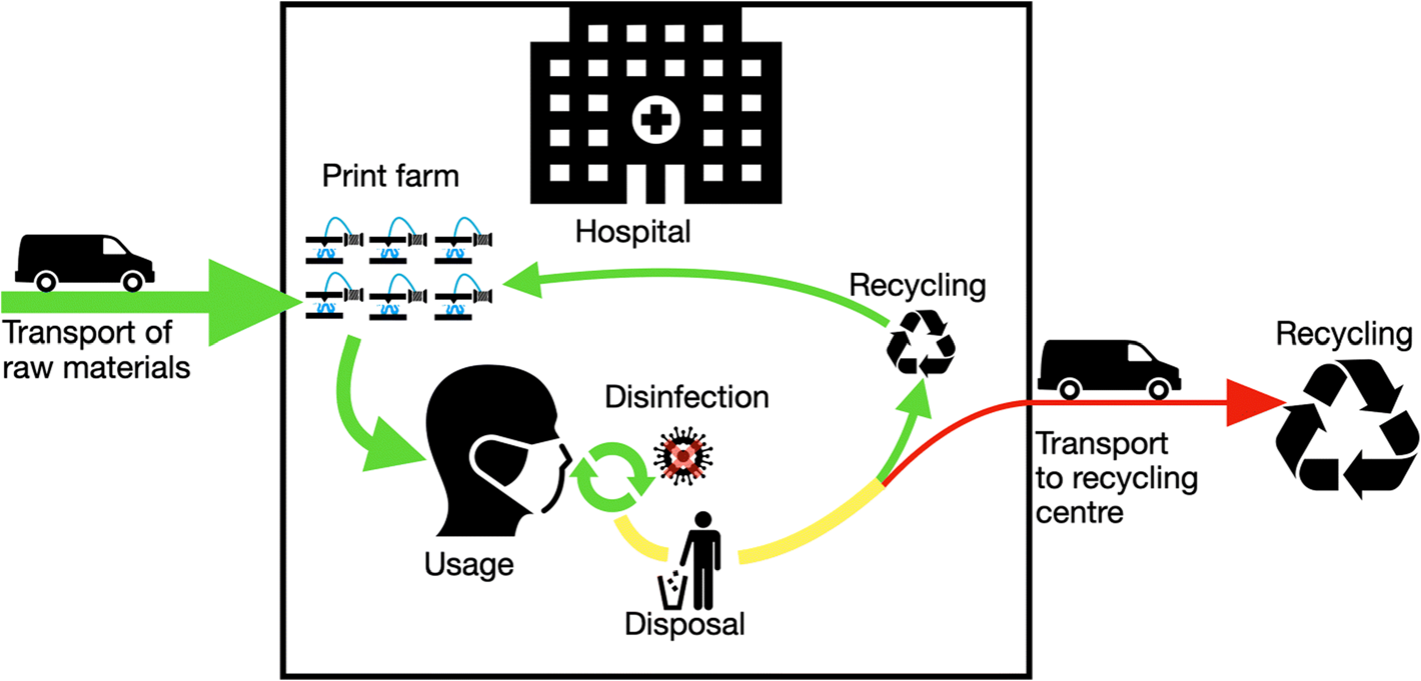
Altered life cycle for a medical device with in-house production and disinfection or recycling after usage. Only the raw material for production will be delivered to the hospital. After production and the subsequent quality assurance, the products are ready for use. After usage, the products undergo disinfection and, if desired, are reused. Otherwise, the waste is recycled or disposed
